# Prevalence of carbapenem-resistant *Acinetobacter baumannii* from 2005 to 2016 in Switzerland

**DOI:** 10.1186/s12879-018-3061-5

**Published:** 2018-04-03

**Authors:** A. Ramette, A. Kronenberg, and the Swiss Centre for Antibiotic Resistance (ANRESIS), A. Burnens, A. Cherkaoui, O. Dubuis, A. Egli, V. Gaia, D. Koch, A. Kronenberg, S. L. Leib, P. Nordmann, V. Perreten, J.-C. Piffaretti, G. Prod’Hom, J. Schrenzel, A. F. Widmer, G. Zanetti, R. Zbinden

**Affiliations:** Institute for Infectious Diseases, University of Bern, Friedbühlstrasse 51, CH-3001 Bern, Switzerland

**Keywords:** Carbapenem resistance, *Acinetobacter baumannii* complex, Surveillance, Epidemiology, Temporal trends, Regional trends

## Abstract

**Background:**

We describe the prevalence of invasive carbapenem-resistant *Acinetobacter* spp. isolated from 2005 to 2016 in different regions of Switzerland.

**Methods:**

Using the Swiss Antibiotic Resistance Centre (anresis) database that includes data from 70% of all hospitalized patients and one third of all ambulatory practitioners in Switzerland, we analysed the number of carbapenem-susceptible and resistant *Acinetobacter* spp. isolated from blood or cerebrospinal fluid, and further described their temporal and regional fluctuations.

**Results:**

From 2005 to 2016, 58 cases of resistant or intermediate strains to carbapenem were observed among 632 cases of invasive *Acinetobacter*. Multivariable analyses indicated that the number of carbapenem-resistant isolates (mean 4.8 ± sd 2.12) and carbapenem resistance rates per region per annum (8.4% ± 13.9%) were low and stable over the studied period. Large fluctuations were observed at the regional level, with e.g. the North East region displaying resistance rates twice as high as that found in other regions.

**Conclusion:**

Despite a relatively stable number of carbapenem-resistant *Acinetobacter* isolates in Switzerland, our results suggest the existence of a diverse pool of *A. baumannii* species in hospital settings, and confirm the implication of carbapenem-resistant *Acinetobacter calcoaceticus*-*Acinetobacter baumannii* (ACB) complex in the vast majority of clinical infections and nosocomial outbreaks with notable regional fluctuations.

**Electronic supplementary material:**

The online version of this article (10.1186/s12879-018-3061-5) contains supplementary material, which is available to authorized users.

## Background

In the Gram-negative, strictly aerobic *Acinetobacter* genus, the species that mostly present a risk as opportunistic human pathogens are *A. baumannii*, *A. nosocomialis, A. pittii*, *A. calcoaceticus*, *A. seifertii* and *A. dijkshoorniae,* which belong to the so-called *Acinetobacter calcoaceticus*-*Acinetobacter baumannii* (ACB) complex. Generally, these four species are not well differentiated at the phenotypic level [1]. They represent the most relevant species clinically, as they may cause serious, difficult-to-treat infections in patients in healthcare settings [1, 2], and are prevalent in the European Union / European Economic Area countries. In contrast, “non-ACB*” Acinetobacter* species generally present lower pathogenicity and are often found in the environment. Yet, uncommon and opportunistic non-ACB *Acinetobacter* species, such as *A. ursingii,* may also cause bloodstream infections [3, 4]. Over the last years, the epidemiological situation has worsened in Europe and worldwide with reports of inter-regional spread and endemic establishment of *A. baumannii* resistant to carbapenems, a last-line group of β-lactam antibiotics used to treat patients infected with multidrug-resistant Gram-negative bacteria [5–8]. Here we report on the temporal and regional analysis of invasive carbapenem-resistant *Acinetobacter* isolated in Switzerland from 2005 to 2016.

## Methods

Antibiotic susceptibility results (AST) were extracted from the database of the Swiss Antibiotic Resistance Centre (anresis.ch) on 8 June 2017. This database includes all AST results from 20 participating laboratories, representing about 70% of all hospitalized patients and one third of all ambulatory practitioners. The laboratories are geographically spread over all Swiss regions and include private, university and general hospital laboratories. These laboratories send all results from routine testing of clinical bacteriology cultures to the anresis database on a regular basis (weekly or monthly). In contrast to other surveillance systems, all antimicrobial resistance results are sent to the anresis database, not restricting the data either to invasive isolates or to specific microorganisms. To allow for higher comparability with international reports, we therefore used the same approach as in the antibiotic surveillance systems of the ECDC (EARS) and of the WHO-Europe (CASEAR), which restrict their analyses to invasive isolates from blood cultures or cerebrospinal fluid. Duplicate entries were removed and only the first date of occurrence of *Acinetobacter* isolation in case of re-infected patients was kept for a given year. Isolates from foreign countries were excluded.

We used the interpreted, qualitative data (SIR) delivered by the participating laboratories, as most microbiological laboratories send only qualitative, interpreted resistance data (SIR). SIR data are not validated by anresis.ch but by the laboratories sending the data. All laboratories participating in anresis.ch are approved and participate in at least one external quality program from NEQAS (https://ukneqas.org.uk/) or from the Quality Control Center Switzerland (http://www.cscq.ch). Isolates are classified as susceptible, non-susceptible or intermediate to at least one of imipenem or meropenem following clinical breakpoints of the given years published in the European Committee on Antimicrobial Susceptibility Testing (EUCAST) guidelines (www.eucast.org) or, according to the Clinical and Laboratory Standards Institute document M31-A3 (CLSI). Most of the participating laboratories switched from CLSI to EUCAST breakpoints between 2011 and 2013.

In addition to SIR data, local laboratories provide accompanying epidemiological information, such as sample location, provider of the sample, patient sex and age-group, but no clinical data about diagnosis, therapy or outcome. Isolate prevalence was modelled via Generalized Poisson regression and resistance rates via logistic regression, with year and regions as explanatory variables. We calculated 95% confidence intervals using robust standard errors for the parameter estimates of the Generalized Poisson regression [9]. Analyses and visualization were done with the R statistical environment (version 3.3.2).

## Results

From 2005 to 2016, a total of 800 invasive *Acinetobacter* isolates were identified in the anresis.ch database, consisting of 707 carbapenem-susceptible and 93 carbapenem-resistant isolates, respectively. After removal of duplicates, 58 resistant or intermediate isolates were identified out of 632 cases (resistance rate 9.2%) over the study period (Table 1). Four out of 58 carbapenem-resistant isolates were isolated from cerebrospinal fluid, the rest from blood cultures. Co-resistance to other antibiotics, such as aminoglycosides (47/55, 86%), trimethoprim-sulfamethoxazole (42/54, 78%) and fluoroquinolones (47/55, 86%), was high, whereas no colistin-resistance was reported for 23 isolates tested. There was a significant increase in the total number of *Acinetobacter* isolations over time (linear regression, F = 21.56, *P* < 0.001, R^2^ = 0.65) with 32.1 isolates per year on average (*t* = 6.39, *P* < 0.001), increasing at a yearly rate of 3.2 new isolates (*t* = 4.64, *P* < 0.001). When ACB complex species were only considered, there was 18.6 isolates per year on average (*t* = 5.62, *P* < 0.001), increasing at 0.98 (*t* = 2.17, *P* = 0.055) new isolates per year. The largest number of resistant isolates belonged to the ACB complex (55/299, 18.4%), while resistance rates were much lower in species not belonging to the ACB complex (1/184, 0.5%). For *Acinetobacter* isolates not typified at species level, 2 out of 149 (1.3%) were carbapenem resistant (Table 1).

**Table 1:** Number and taxonomic affiliation of invasive *Acinetobacter* isolates from 2005 to 2016 in Switzerland

				Taxonomic affiliation^a^									
	Susceptibility rates			ACB complex	Non-ACB complex								Isolates not typified
	*n*	S (%)	R (%)	*A. baumannii* *A. calcoaceticus* *A. nosocomialis* *A. pittii*	*A. ursingii*	*A. lwoffii*	*A. junii*	*A. johnsonii*	*A. haemolyticus*	*A. radioresistens*	*A. baylyi*	*A. parvus*	
2005	37	34 (91.9)	3 (8.1)	2/20 ^a^	0/0	0/9	0/0	0/0	0/1	0/0	0/0	0/0	1/4
2006	48	44 (91.7)	4 (8.3)	4/27	0/0	0/7	0/1	0/0	0/0	0/0	0/0	0/0	0/9
2007	32	32 (100.0)	0 (0.0)	0/16	0/0	0/8	0/1	0/1	0/0	0/0	0/0	0/0	0/6
2008	48	43 (89.6)	5 (10.4)	5/13	0/0	0/12	0/1	0/0	0/0	0/0	0/0	0/0	0/17
2009	35	31 (88.6)	4 (11.4)	4/11	0/1	0/6	0/0	0/0	0/0	0/0	0/0	0/0	0/13
2010	61	56 (91.8)	5 (8.2)	5/22	0/1	0/14	0/3	0/0	0/0	0/1	0/0	0/0	0/15
2011	53	49 (92.5)	4 (7.5)	4/23	0/4	0/5	0/1	0/0	0/0	0/0	0/0	0/0	0/16
2012	53	45 (84.9)	8 (15.1)	8/17	0/2	0/10	0/3	0/0	0/1	0/0	0/0	0/1	0/11
2013	54	47 (87.0)	7 (13.0)	7/16	0/4	0/9	0/1	0/1	0/1	0/2	0/0	0/0	0/13
2014	75	69 (92.0)	6 (8.0)	6/26	0/7	0/10	0/0	0/3	0/1	0/3	0/0	0/0	0/19
2015	64	57 (89.1)	7 (10.9)	5/25	1/4	0/12	0/2	0/0	0/2	0/0	0/0	0/0	1/12
2016	72	61 (93.8)	5 ( 6.9)	5/28	0/10	0/6	0/8	0/2	0/0	0/0	0/1	0/0	0/12
Total	632	574 (90.8)	58 (9.2)	55/244	1/33	0/108	0/21	0/7	0/6	0/6	0/1	0/1	2/147

^a^The first and second numbers correspond to the number of resistant and number of susceptible isolates, respectively. Overall, 485 of 632 (76.7%) of the isolates were characterized at the species level

Generalized Poisson regression of the number of carbapenem-resistant isolates on year (from 2005 to 2016) and across 8 Swiss regions indicated that year has no significant effect on the average of 4.83 (sd 2.13) total *Acinetobacter* isolates per year or on the number of ACB isolates (average 4.58 sd 2.11), with incident rate ratios [IRR] of 1.00 (95% CI 0.96–1.04, *P* > 0.050) for both ACB and non-ACB isolates (Additional file 1: Table S1). The North East region, with a total of 24 resistant *Acinetobacter* (22 of which were ACB) isolations from 2005 to 2016, was significantly above all other regions in terms of number of resistant isolates, e.g. the comparison with Centre East (2 isolates, both ACB) taken as the reference region yielded IRR = 2.00 (1.35–2.98), *P* < 0.001 (for ACB only, values were IRR = 2.19 (1.46–3.28), *P* < 0.001). Other regions with 4.9 (sd 2.5) carbapenem-resistant *Acinetobacter* isolates on average were not significantly different from Centre East (*P* > 0.05).

**Fig. 1:**
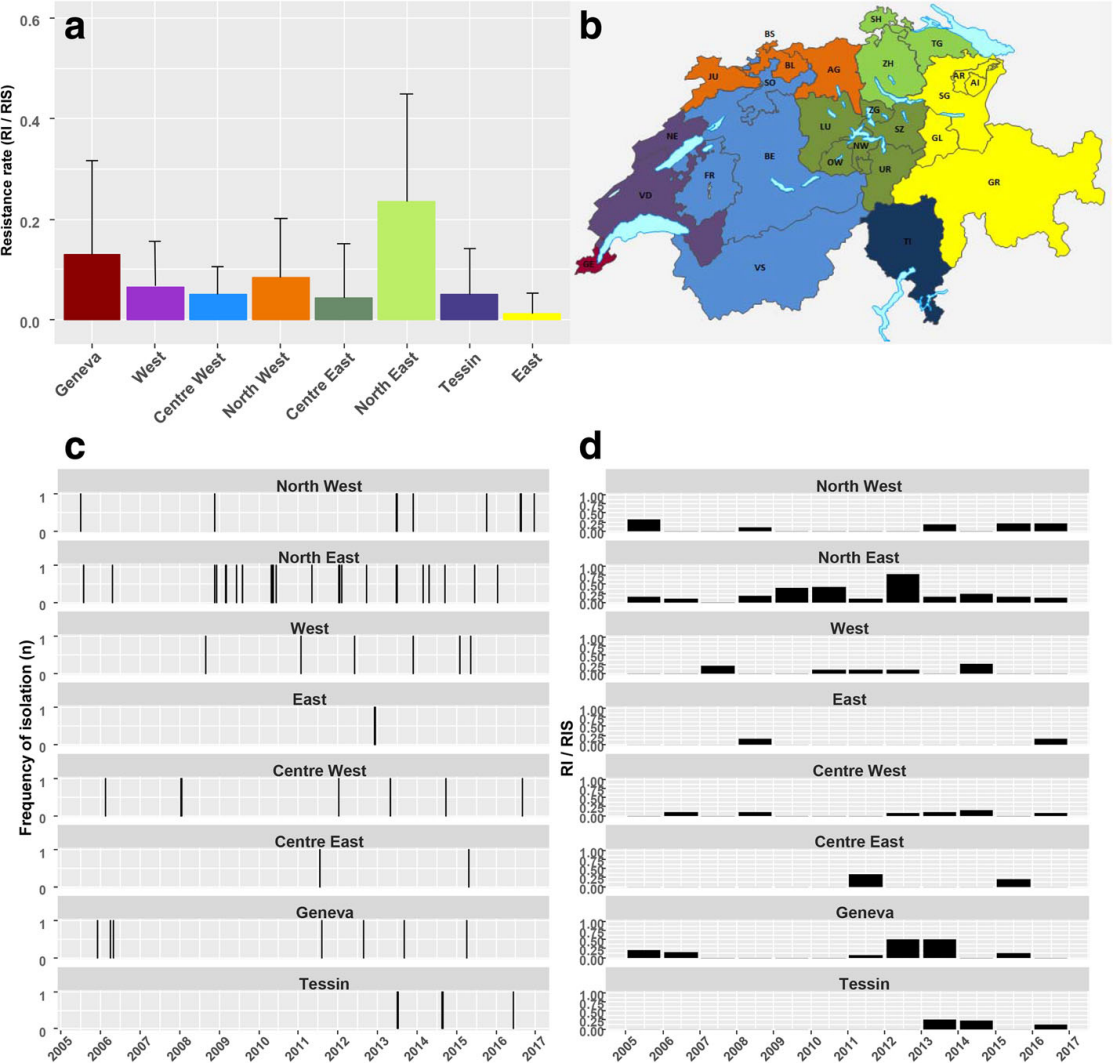
**a** *Acinetobacter* resistance rates (number of resistant isolates compared to total number of isolates) per region from 2005 to 2016 in Switzerland. Standard deviation bars represent annual fluctuations per region. **b** Map of the Swiss regions defined in this study. The original map template, which was made freely available by the Federal Statistical Office (https://www.bfs.admin.ch/bfs/en/home/statistics/regional-statistics/maps.html), was modified by the authors. **c** Frequency of isolation of resistant *Acinetobacter* across Swiss regions from 2005 to 2016 (daily resolution). **d** *Acinetobacter* resistance rates per year and per region in Switzerland

Those trends were confirmed when examining resistance rates per year per region (Fig. 1a, b), which reached 8.4% (sd 13.9%) on average, and for ACB isolates 15.4% (sd 26.1%). There was no significant temporal trend in resistance rates (for all *Acinetobacter* isolates, OR 1.06 (0.98–1.16), *P* = 0.155; for ACB isolates only, OR 1.08 (0.99–1.19), *P* = 0.096). Only the North East region (resistance rates for all *Acinetobacter* isolates of 23.4% sd 22%; for ACB isolates, 32.9% sd 30.7%) had higher rates on average (OR 6.75 (1.88–43.32), *P* = 0.012; for ACB isolates, 4.95 (1.24–33.3) *P* = 0.045) than region Center East (resistance rate 4.4% sd 10.8%; for ACB isolates, 11.1% sd 29.6%). Other regions had resistance rates ranging from 1.3 to 13.0% (2.1 to 19.8% for ACB isolates), which were not significantly different from that of Center East (*P* > 0.05).

At a more detailed level of analyses (daily resolution, Fig. 1c; and resistance rate per region per year, Fig. 1d), number of cases and carbapenem-resistance rates associated with *Acinetobacter* isolates displayed large temporal and regional disparities, both displaying patterns reminiscent of outbreaks: the detailed analysis of the increase in 2010 observed in the North East region was highly suggestive of an outbreak because 5 resistant *Acinetobacter* isolations, all belonging to ACB, occurred for the whole region within six weeks in the same intensive care unit of burn patients.

We tested the effect of the variation in laboratories contributing to the anresis database on our conclusions by using the data provided by the 14 laboratories that always sent data from 2005 onwards. This sensitivity analyses showed that the number of resistant isolates and resistance rates over year and region did not change qualitatively as compared to the results obtained with the complete dataset (Additional file 1**:** Table S1): The number of total resistant isolates in the sensitivity analyses did not significantly increase over time and the North East region displayed higher number of isolates than any other region, considering all Acinetobacter isolates or only ACB isolates (Additional file 1: Table S1). Similarly, the sensitivity analysis of the factors affecting resistance rates also confirmed that there was no significant temporal changes for all *Acinetobacter* isolates and when considering ACB isolates only (the temporal trend was marginally significant for ACB with *P* = 0.042; Additional file 1: Table S1), and that the North East region had higher rates on average than other regions (resistance rates in the sensitivity analyses ranging from 1.0 to 11.1 and 0 to 24.0% for ACB isolates).

## Discussion

A publication by the ECDC of an article entitled “Carbapenem-resistant *Acinetobacter baumannii* in healthcare settings” [8], concluded that “there has been an overall increase in carbapenem-resistant *Acinetobacter baumannii* in Europe, especially in countries of lower prevalence.” Using the database of the Swiss Antibiotic Resistance Centre (anresis.ch), which includes all AST results representing about 70% of all hospitalized patients and one third of all ambulatory practitioners in Switzerland, we report on the first nationwide surveillance study on invasive carbapenem-resistant *Acinetobacter* isolated from 2005 to 2016 in Switzerland. Importantly, we observed that the resistance rate has stabilized yearly at 8.4%, with large temporal and regional disparities, but without increase over time.

Our results indicate the existence of a diverse pool of *A. baumannii* and related species in hospital settings in Switzerland, as also observed in France [10]. We confirmed the implication of carbapenem-resistant ACB complex isolates in the vast majority of clinical infections and nosocomial outbreaks that involved *Acinetobacter* isolates, as observed previously in Germany over a 5-year period [11]. Our findings were also in agreement with the known higher risk of infection by *A. baumannii* in burn patients in hospital settings, as already reported in Switzerland [12] and in other countries [13, 14].

The strengths of our study are that analyses were based on data collected regularly and systematically over 10 years, covering a large proportion of hospitalized patients in Switzerland. Limitations of our study include the fact that there are no mandatory Swiss guidelines for antibiotic resistance testing. Yet the Swiss Society of Microbiology encourages the use of EUCAST breakpoints and provides recommendations on their website (http://www.swissmicrobiology.ch). Nevertheless individual laboratories are free to use other guidelines and methods than EUCAST. Although most laboratories initially based their testing on CLSI guidelines, they changed to EUCAST guidelines between 2011 and 2013, with in general an increased use of automated systems over the years. Therefore identification methods may differ between different laboratories. Another limitation is the fact that clinical isolates were classified as “susceptible”, “intermediate”, or “resistant” based on clinical breakpoints. As such, quantitative resistance data are not available for most isolates. This classification indicates the likelihood of a therapeutic success (e.g. dosing, method and route of administration, pharmacokinetic and pharmacodynamics) with a certain antibiotic and thus helps the attending physician to select the best possible treatment. The use of different clinical breakpoints (e.g. EUCAST vs. CLSI) or changing breakpoints over time may therefore influence the results. However with the change from CLSI breakpoints to EUCAST breakpoints (as performed by most of the anresis-laboratories between 2011 and 2013) we would expect a methodology-related increase in resistance rates during this time period, as breakpoints for susceptibility by disc diffusion in EUCAST are higher than in CLSI 2009–2011 (imipenem ≥23 vs. ≥16, meropenem ≥21 vs. ≥16, respectively; [15]). This consideration  strengthens further our conclusion that carbapenem resistance in *Acinetobacter* did not increase over time in Switzerland.

## Conclusions

Our analyses which cover both multiple years and multiple regions conjointly highlight the usefulness of surveillance approaches that integrate different temporal and spatial resolution levels. Therefore, further surveillance efforts are needed to detect and control *Acinetobacter* outbreaks, and to limit the endemic establishment of resistant isolates in additional health facilities and across EU regions.

## Additional file

Additional file 1: **Table S1.** Sensitivity analyses of temporal and regional trends in *Acinetobacter* and in ACB (number of isolates, resistance rates). We compared the effects of year and region on the number of resistant isolates and on resistant rates by considering data sent either by all laboratories or by laboratories that regularly sent data since 2005. (DOCX 22 kb)

## Abbreviations

ACB: *Acinetobacter calcoaceticus*-*Acinetobacter baumannii*
AST: Antibiotic susceptibility test
CI: Confidence interval
IRR: Incident rate ratio
OR: Odds ratio
sd: Standard deviation
