# Supplementary material for: Prevalence of carbapenem-resistant Acinetobacter baumannii from 2005 to 2016 in Switzerland
Source: BMC Infect Dis. 2018 Apr 3;18:159. doi: 10.1186/s12879-018-3061-5 (PMC5883518; doi:10.1186/s12879-018-3061-5)
Supplement: Supplementary file 1 — Table S1. Sensitivity analyses of temporal and regional trends in Acinetobacter and in ACB (number of isolates, resistance rates). We compared the effects of year and region on the number of resistant isolates and on resistant rates by considering data sent either by all laboratories or by laboratories that regularly sent data since 2005. (DOCX 22 kb) [file 12879_2018_3061_MOESM1_ESM.docx]

| Supplementary Table 1. Sensitivity analyses of temporal and regional trends in *Acinetobacter* and in ACB (number of isolates, resistance rates) | | | | | | | | | | |
| --- | --- | --- | --- | --- | --- | --- | --- | --- | --- | --- |
|  | All laboratories | | | | | | Laboratories sending data since 2005 | | | |
|  | All *Acinetobacter* | | | Only ACB isolates | | | All *Acinetobacter* | | Only ACB isolates | |
| **Number of laboratories** | 23 | | | 22 | | | 14 | | 14 | |
| **Total number of isolates** | 632 | | | 299 | | | 495 | | 236 | |
| **Number of resistant isolates** | 58 | | | 55 | | | 58 | | 48 | |
| **Global resistance rate** | 9.2% | | | 18.4% | | | 11.7% | | 20.3% | |
| **Mean number of isolates sent per year per region** | 4.83 (2.13) | | | 4.58 (2.11) | | | 4.25 (1.71) | | 4.0 (1.71) | |
| **Factors affecting the number of resistant isolates per year per region^a^** | | | | | | | | | | |
|  | IRR (95% CI) | P value | | IRR (95% CI) | P value | | IRR (95% CI) | P value | IRR (95% CI) | P value |
| Year | 1.00 (0.96-1.04) | 0.979 | | 1.00 (0.95-1.04) | 0.909 | | 1.00 (0.96-1.04) | 0.979 | 0.99 (0.94-1.04) | 0.688 |
| Centre East | 1.00 (-) | - | | 1.00 (-) | - | | 1.00 (-) | - | 1.00 (-) | - |
| Centre West | 1.00 (0.94-1.06) | 0.979 | | 1 (0.93-1.07) | 0.910 | | 1.00 (0.94-1.06) | 0.979 | 0.98 (0.90-1.08) | 0.727 |
| East | 1.00 (0.96-1.04) | 0.979 | | 1.00 (0.95-1.04) | 0.910 | | NA | NA | NA | NA |
| Geneva | 1.17 (0.91-1.50) | 0.226 | | 1.16 (0.91-1.48) | 0.240 | | 1.17 (0.91-1.50) | 0.226 | 1.16 (0.87-1.55) | 0.308 |
| North East | 2.00 (1.35-2.98) | 0.001 | | 2.19 (1.46-3.28) | <0.001 | | 2.00 (1.35-2.98) | 0.001 | 2.39 (1.60-3.58) | <0.001 |
| North West | 1.00 (0.97-1.03) | 0.979 | | 1.00 (0.95-1.05) | 0.912 | | 1.00 (0.97-1.03) | 0.979 | 0.97 (0.84-1.13) | 0.716 |
| Tessin | 1.00 (0.95-1.05) | 0.979 | | 1.00 (0.95-1.06) | 0.910 | | 1.00 (0.95-1.05) | 0.979 | 1.35 (0.90-2.03) | 0.145 |
| West | 1.17 (0.90-1.51) | 0.245 | | 1.16 (0.89-1.52) | 0.259 | | 1.17 (0.90-1.51) | 0.245 | 1.31 (0.84-2.05) | 0.227 |
| **Mean resistant rate:** |  | | |  | | |  | |  | |
| per region (sd) | 8.4% (6.98%) | | | 15.3% (9.61%) | | | 8.3% (7.42%) | | 15.3% (10.23%) | |
| per region per year (sd) | 8.4% (13.9%) | | | 15.4% (26.1%) | | | 8.3% (14.0%) | | 15.3% (27.5%) | |
| **Factors affecting resistant rates per year per region^b^** | | | | | | | | | | |
|  | OR (95%CI) | | P value | OR (95%CI) | | P value | OR (95%CI) | P value | OR (95%CI) | P value |
| Year | 1.06 (0.98-1.16) | | 0.155 | 1.08 (0.99- 1.19) | | 0.096 | 1.07 (0.98- 1.17) | 0.134 | 1.11 (1.01-1.22) | 0.041 |
| Centre East | 1.00 (-) | | - | 1.00 (-) | | - | 1.00 (-) | - | 1.00 (-) | - |
| Centre West | 1.27 (0.28-8.91) | | 0.780 | 1.27 (0.26- 9.34) | | 0.782 | 1.40 (0.31- 9.86) | 0.686 | 1.41 (0.28-10.5) | 0.699 |
| East | 0.43 (0.02-4.70) | | 0.501 | 0.36 (0.02- 4.15) | | 0.425 | NA | NA | NA | NA |
| Geneva | 2.51 (0.57-17.41) | | 0.265 | 2.12 (0.45-15.49) | | 0.383 | 2.38 (0.52-16.84) | 0.305 | 2.08 (0.41-15.8) | 0.411 |
| North East | 6.75 (1.88-43.32) | | 0.012 | 4.95 (1.24-33.34) | | 0.045 | 7.01 (1.94-45.11) | 0.011 | 4.89 (1.19-33.4) | 0.050 |
| North West | 2.05 (0.47-14.20) | | 0.383 | 1.60 (0.32-11.82) | | 0.592 | 2.58 (0.52-18.72) | 0.275 | 2.70 (0.44-22.4) | 0.301 |
| Tessin | 1.45 (0.27-10.78) | | 0.677 | 0.87 (0.15- 6.74) | | 0.878 | 1.41 (0.26-10.50) | 0.701 | 0.78 (0.13-6.12) | 0.788 |
| West | 1.76 (0.40-12.17) | | 0.492 | 1.50 (0.31-10.92) | | 0.637 | 1.36 (0.25-10.16) | 0.729 | 1.06 (0.18-8.45) | 0.948 |
| ^a^IRR: incident rate ration from Poisson regression; 95% confidence intervals for the estimates are provided in parentheses ^b^OR: Odds Ratio from logistic regression; 95% confidence intervals for the estimates are provided in parentheses. “-“category was used as reference category for factor “region”; “NA” not available due to the lack of data. | | | | | | | | | | |
